# Advertising

**Published:** 1891-10

**Authors:** 


### Advertising.

"PRIVATE AND CONFIDENTIAL."

"*Dear Sir:*—May I venture upon a suggestion, by the adoption of which, mutual advantage should result, viz:—that I shall be happy to allow you a commission of 20 per cent. upon any fresh professional business that you may be the means of introducing to me, with 5 per cent. upon all renewals therefrom, and 5 per cent. upon any collateral introduction. My fees are as follows: 10s. 6d. for each operation, including stopping, extractions, etc., etc. Sets of teeth, 10 guineas, 15 guineas and 20 guineas. Servants and poor persons half fees.

I am, dear sir,

Etc., Etc."

So runs a notice which has come into our hands. It seems to us, there may perfectly well be two opinions as to advertising, in an open and above board manner, but surely there can be only one, and that decidedly adverse to such a way of endeavoring to rake together patients. We do not know in the least what was the result of this miserable supplication and confession of being out of work, but certainly if a medical man has any claim at all to be looked upon as a professional man, he would promptly look on such a communication as a personal insult. We notice that the writer of this holds a hospital appointment; it would be curious to know whether the other members of the staff have had this brought to their knowledge, and if so, what notice they are going to take of it.

The question of advertising is a very large one. Every man must become known if he would gather around himself a practice; he must advertise himself in every way that is legitimate; but it is around this point, what is, and what is not legitimate; that the battle wages. We would not, of course, like to take upon ourselves the task of laying down the law, of drawing a hard and fast line which should divide that which is just, from that which is unjust, but still there are certain things which can be condemned without a second thought, there are others which the more high-minded would not do, but which only raises an amused smile when we hear of their being done; there are, again, other matters concerning the correctness of which is so knotty a point that one's own views are apt to vary from day to day, as one's moods and fancies change.

Surely, there is nothing more amusing than to see a man endeavoring to make himself known, by writing on all sorts of subjects to all sorts of papers; notice how prominently the address is placed, how carefully the qualifications are attached, and what a lot is made of a little. Then again, how eagerly some set to work to write a book, a collection of all that is old, but flavored with nothing that is new. Here again notice the preface. The first personal pronouns almost elbow one another, so closely do they crowd, while to crown them all comes the signature with the flourish of an adept, likewise not forgetting to append the address. Success in life is to a large extent due to judicious advertising. Note how often a very third rate man does, in the end, become of some importance. The importance of this element of push in attaining success has been fully recognized, and must ever be regarded as perfectly legitimate. Quite different, however, are those obnoxious advertisements, which, but too frequently disfigure the public press, not to mention the dropping of pamphlets down the area. Perhaps if all that these advertisements state was strictly true, if the inducements offered were carried out, in the spirit as in the letter, one might even to some extent pardon these practices, alas! we know only too well that they are not. Take the question of show cases. We suppose the work displayed in so hideous a manner is meant to be taken as that of the man who is waiting at the shop door to inveigle the unwary into his meshes. But is it? If so, what means the advertisement one finds sometimes as to "specimen cases" for sale. The fact is, such cases are often simply bought for the purpose of display, and is as much like the work which the advertiser supplies his patients with, as is a brick-bat like a Dutch cheese. Take again the question of advertising low fees as an inducement to patients to go to a particular man, does any one pretend for a moment that these fees are adhered to? Is it not a matter of fact that every opportunity is taken, and excuse made for asking a higher fee? On the grounds of common honesty we ask, should not such advertisements be condemned?

But if we look at this question from the point of view of professional ethics, nothing but blame can attach to such advertising. We do not know what are the motives of such men, whether they have any pride in their work, any wish to be a member of an honorable profession, or any other ambition beyond the earning of a few pounds. We do not know what is the frame of mind which possesses such men, whether if they were equally well paid, they would as soon be shoe-blacks, crossing-sweepers or perhaps sorters on a muck-heap. There are some things one can never fathom, and we could never understand the man who ceases to take pride in his work, cast himself loose from all honorable associations, and proceed simply to make what he can by fair means, or foul. It is an unsavory matter one is glad to turn away from, and in charity hope that things are not always what they seem.—*British Jour. of Den. Science.*

